# The Probiotic *Pediococcus acidilactici* in the Feed of Salmonids: A Strategy to Improve Reproductive Parameters

**DOI:** 10.3390/ani15111659

**Published:** 2025-06-04

**Authors:** Rommy Díaz, Doris Carrasco, John Quiñones, Ailín Martínez, Gastón Sepúlveda, Isabela Pérez-Núñez, Rodrigo Huaiquipán, David Cancino-Baier, Jorge F. Beltrán, Jorge G. Farías, Erwin A. Paz, Néstor Sepúlveda

**Affiliations:** 1 Facultad de Ciencias Agropecuarias y Medioambiente, Universidad de La Frontera, Temuco 4780000, Chile; john.quinones@ufrontera.cl (J.Q.); david.cancino@ufrontera.cl (D.C.-B.); nestor.sepulveda@ufrontera.cl (N.S.); 2 Laboratorio de Producción Animal y Centro de Tecnología e Innovación en Calidad de la Carne (CTI-Carne), Universidad de La Frontera, Temuco 4780000, Chile; a.martinez26@ufromail.cl (A.M.); g.sepulveda10@ufromail.cl (G.S.); i.perez04@ufromail.cl (I.P.-N.); r.huaiquipan01@ufromail.cl (R.H.); 3 Departamento de Ciencias Veterinarias y Salud Pública, Facultad de Recursos Naturales, Universidad Católica de Temuco, Temuco 4780000, Chile; doris.carrasco@uct.cl; 4 Programa de Doctorado en Ciencias Mención Biología Celular y Molecular Aplicada, Universidad de La Frontera, Temuco 4780000, Chile; 5 Programa de Doctorado en Ciencias Agroalimentarias y Medioambiente, Universidad de La Frontera, Temuco 4780000, Chile; 6 Departamento de Ingeniería Química, Facultad de Ingeniería y Ciencias, Universidad de La Frontera, Temuco 4780000, Chile; beltran.lissabet.jf@gmail.com (J.F.B.); jorge.farias@ufrontera.cl (J.G.F.); 7 UWA Institute of Agriculture, The University of Western Australia, Perth, WA 6009, Australia; erwin.pazmunoz@uwa.edu.au

**Keywords:** salmon broodstock, sperm quality, fertility, probiotics, *Pediococcus acidilactici*, embryo

## Abstract

This study investigated the effects of dietary supplementation with the probiotic *Pediococcus acidilactici* on the reproductive performance and offspring viability of male Atlantic salmon (*Salmo salar*). A total of 48 mature males were divided into three groups—Group A (control, no probiotic), Group B (60-day supplementation), and Group C (120-day supplementation). The fish were kept in 10 m^3^ tanks at a constant temperature of 9 °C under a natural photoperiod and evaluated for multiple reproductive and physiological parameters. The results demonstrate that Group C exhibited significantly higher gonad weight, gonadosomatic index (GSI), and sperm concentration than Groups A and B. The blood biochemistry revealed reduced bilirubin and alanine aminotransferase (ALT) levels in Group C; however, values were within normal reference ranges for the species in all groups. While sperm quality parameters such as membrane integrity and motility showed no significant differences, fertility and embryo viability were significantly higher in Group C across all three developmental stages (180, 300, and 380 accumulated thermal units, ATUs). Additionally, Group C showed lower rates of embryonic malformation and mortality at later stages of development. Statistical analyses confirmed a significant interaction between treatment duration and embryo viability, with the 120-day probiotic treatment (Group C) producing the best outcomes. These findings support the hypothesis that *P. acidilactici* supplementation positively influences male reproductive parameters and improves embryo quality and survival in Atlantic salmon.

## Simple Summary

This study evaluated the potential benefits of dietary supplementation with the probiotic *Pediococcus acidilactici* for the reproductive performance of male Atlantic salmon and the viability of their offspring. Male salmon were fed the probiotic for either 60 or 120 days and compared with a control group that received no supplementation. Males supplemented for 120 days demonstrated enhanced reproductive traits, including increased gonad weight and sperm concentration. Although no significant differences in sperm quality parameters were observed among the groups, the extended probiotic treatment resulted in higher embryo survival rates and a lower incidence of developmental deformities. These findings suggest that dietary supplementation with probiotics may positively influence reproductive outcomes and offspring quality in farmed Atlantic salmon.

## 1. Introduction

Fish is an important source of protein and nutrients, including vitamins (A, D, and B-complex), minerals (such as calcium, iron, and selenium), and very-long-chain polyunsaturated fatty acids (VLC-PUFAs) [1,2]. These nutrients play a fundamental role in physiological processes such as child nutrition, brain function, and nervous system development, collectively promoting human health [3]. Over the past decades, the drastic decline in fishery production has made aquaculture the primary global source of fish. Currently, aquaculture supplies more than half of the world’s fish consumption, highlighting its crucial role in ensuring food security and meeting the nutritional needs of the population [4,5,6,7,8]. The 2024 edition of The State of World Fisheries and Aquaculture reported that in 2022, aquaculture surpassed capture fisheries as the main producer of aquatic animals, contributing 51% of total aquatic animal production [9].

In this context, ensuring the sustainability and productivity of aquaculture systems becomes essential, with reproduction standing out as one of the most critical components of the aquaculture value chain. This process involves optimizing various factors such as welfare and health, nutrition, sexual maturation, gamete production, and efficient spawning management [10,11]. However, captive broodstocks often face a higher risk of reproductive dysfunctions due to hormonal imbalances and environmental constraints [12]. Common reproductive deficiencies include the production of poor-quality gametes, inefficient or weak spawning, reduced larval growth rates—particularly during the yolk sac absorption phase and early exogenous feeding—and high fry mortality. These dysfunctions often result from direct or indirect alterations in gametes and the endocrine system, stress induced by captivity, suboptimal spawning conditions, and nutritional deficiencies in broodstock diets [13,14,15].

As in other living organisms, welfare and health are crucial factors in fish reproduction. This principle has driven the development of pharmaceuticals and vaccines aimed at strengthening the broodstock immune system, thereby enhancing reproductive capacity and overall well-being [16]. Recent advances in nanotechnology, epigenetics, and dietary supplements have shown great potential in improving not only reproduction but also overall fish health [10,17,18]. Additionally, fish health is influenced by extrinsic factors such as nutrition and intrinsic factors such as the microbiome, whose relationship with reproductive processes has gained increasing research interest [19].

In recent years, probiotics have emerged as a sustainable alternative to synthetic antibiotics and chemical products [20,21,22]. These beneficial microorganisms have demonstrated their ability to improve fish health and reproductive performance by stabilizing intestinal microbial communities, activating key hormones, and enhancing gene transcription. These effects translate into significant improvements in fertilization, hatching, and survival rates, promoting overall broodstock well-being [1]. In females, probiotic administration can induce the response of incompetent follicles (stage IIIa) to the maturation-inducing hormone (MIH) during the maturation period, as well as alter the chemical composition of oocytes, enhancing vitellogenic development and improving reproductive quality [23]. Despite these advancements, there are still unexplored research areas, such as the effects of probiotics on reproduction, maturation, and fecundity, which require a more comprehensive approach to fully understand their impact and potential in aquaculture [24].

Salmonids are among the most commercially successful aquaculture species worldwide. Norway and Chile are the leading global producers of salmonids, accounting for approximately 53% and 30% of global salmon and rainbow trout production, respectively [25]. The trade of salmon and trout has shown consistent growth, with an average annual increase of 10.4% in value. In 2022, global exports of salmon and trout reached a value of USD 38 billion, with Norway and Chile leading the export market, recording historically high revenues driven by strong global demand, rising prices, and greater value-added products [26]. As a result, salmon, particularly farmed Atlantic salmon, has become a key species contributing to the expansion of global aquatic product trade in recent decades [26]. Beyond its economic importance, salmon is a significant source of long-chain omega-3 polyunsaturated fatty acids (n-3 LC-PUFAs), particularly eicosapentaenoic acid (EPA) and docosahexaenoic acid (DHA), which are widely recognized for their positive impact on human health [27]. During early developmental stages, salmon diet plays a crucial role in improving survival, health, growth, and overall performance, underscoring the importance of proper nutrition from the earliest life stages [28].

Several studies have demonstrated that incorporating probiotics into fish diets from early life stages, such as larvae, is cost-effective, as it reduces disease burden in later developmental stages [21]. Probiotics are live microorganisms that, when ingested, provide significant health benefits by balancing intestinal microbiota. These microorganisms, including bacteria and yeasts, create a favorable environment in the digestive tract by inhibiting the growth of harmful microbes and promoting beneficial ones [29,30,31]. The effects of probiotics depend on the strain used and the individual characteristics of each organism [32,33]. Some strains produce organic acids, enzymes, and antimicrobial substances that inhibit pathogenic bacterial growth, contributing to intestinal homeostasis [34]. Additionally, probiotics interact with immune cells in the intestine, stimulating immune responses through gene activation and cytokine production [35].

In aquaculture, probiotic applications have been studied in various fish species with promising results. In eels, *Lactobacillus rhamnosus* treatment increased sperm volume and motility and induced transcriptomic changes suggesting enhanced spermatogenesis [36]. In zebrafish, dietary supplementation with *Lactobacillus rhamnosus* promoted ovarian development and significantly increased total egg count [37]. Notably, *Pediococcus acidilactici* has been shown to improve reproductive performance in zebrafish by increasing gonadosomatic index, absolute fecundity, relative fecundity, and hatching rate [38]. Similar findings have been reported in goldfish (*Carassius auratus*), where diets supplemented with *Pediococcus acidilactici* enhanced sperm motility, absolute fecundity, and fertilization rates compared to control groups [39].

In salmonids, lactic acid bacteria have been successfully used to increase intestinal bacterial diversity, contributing to a more balanced microbiome [31]. Additionally, probiotic baths with *Aliivibrio fischeri* have been shown to reduce ulcer incidence and improve growth rates in this species [40]. *Pediococcus acidilactici* has demonstrated beneficial effects in various species, enhancing growth performance, intestinal health, and immune responses, making it a valuable dietary supplement [41,42,43,44]. However, there is limited research on the effects of dietary probiotic supplementation on reproductive parameters in salmonids. We hypothesize that supplementation with *Pediococcus acidilactici* improves the reproductive performance of salmonids by promoting the physiological health of these species. Therefore, this study aimed to analyze the effect of *Pediococcus acidilactici* supplementation on the reproductive performance of salmonids, using Atlantic salmon (*Salmo salar*) as a model.

## 2. Materials and Methods

### 2.1. Animal Handling and Study Conditions

This study was conducted in accordance with the ethical guidelines of the Scientific Ethical Committee of the Universidad de La Frontera, Temuco, Chile (Certificate No. 103/19). The research was carried out during autumn at a commercial salmon farm in Curarrehue, La Araucanía Region, Chile (39°23′31.1″ S, 71°40′52.4″ W), coinciding with the natural reproductive cycle of Atlantic salmon (*Salmo salar*). In Chile, salmon farming follows a two-phase system. Juveniles are produced in freshwater hatcheries, where fertilized eggs are incubated, and fry are reared until they reach the smolt stage (80–150 g). These smolts are then transferred to marine net pens in the southern coastal regions of Chile, where they are cultured for 12–18 months until reaching a size of 3–6 kg.

For this study, a total of 48 male broodstock, approximately 4 to 5 years old, with an average weight of 7.77 ± 1.4 kg and an average total length of 79.3 ± 2.1 cm, were selected. These values are within the typical size range for Atlantic salmon at the studied stage, as male *Salmo salar* in their reproductive phase commonly range between 5 and 9 kg, and their length typically varies from 70 to 85 cm. These fish were in the final stages of sexual maturation, having reached sexual maturity between 3 and 5 years of age. At this stage, males exhibited characteristic secondary sexual traits, including kype formation (an enlarged lower jaw) and changes in body coloration, which are indicators of sexual maturity. Gonadal maturation was confirmed through abdominal palpation, and only males showing fluent milt were included in the study. In the Southern Hemisphere, the reproductive season generally peaks during the autumn, with gonadal development and spermiation occurring naturally during this period. The study was conducted during the natural spawning season, when males were ready to release sperm. The broodstock were maintained in 10 m^3^ tanks under a natural 12 h photoperiod and at a stable temperature of 9 °C in a flow-through system with dissolved oxygen levels of 9 ± 1 mg O_2_ L^−1^ (98% saturation). To ensure optimal environmental conditions, these parameters were monitored and recorded daily.

### 2.2. Experimental Design

The fish (*n* = 48) were evenly divided into three experimental groups (A, B, and C) and placed into three separate tanks, where they underwent a 20-day acclimation period. The fish were fed a commercial diet (BioMar, Puerto Montt, Chile) containing 45.5–47% protein, 23.5–25% fat, a maximum moisture content of 10%, and a maximum ash content of 12%. The feed was stored under conditions recommended by the manufacturer to ensure the viability of the probiotic. Specifically, it was kept in a dry and well-ventilated environment, protected from direct sunlight, and maintained at temperatures below 25 °C. The feed was administered twice daily throughout the experiment. Group A (control) was fed the standard diet without probiotic inclusion for 120 days. Groups B and C were fed the standard diet supplemented with the probiotic *Pediococcus acidilactici* MQ 18/5 (Bactocell^®^, Lallemand Animal Nutrition, Blagnac, France) at a concentration of 10^6^ colony-forming units per gram (CFU g^−1^). Group B was fed the standard diet without probiotics for the first 60 days, followed by the supplemented diet for the remaining 60 days (the minimum supplementation time recommended by the manufacturer). Group C received the probiotic-supplemented diet for the entire 120-day period, to assess whether a longer supplementation period is necessary to observe effects in adult fish. At the end of the supplementation period, various physiological and reproductive parameters were evaluated. Biometric measures (weight and length), blood biochemical parameters, semen volume, sperm concentration, and the gonadosomatic index (GSI) were measured. Additionally, to assess the effects of probiotics on gamete quality, sperm motility parameters, membrane integrity, mitochondrial functionality, and oxidative stress indicators were analyzed. Finally, fertility trials and assessments of embryo viability and quality were conducted to determine the influence of probiotic supplementation on offspring quality.

### 2.3. Biometric Measures

At the end of the assay, the males were euthanized using a lethal overdose of anesthetic by immersion in a 50-liter tank containing 500 mg/L of MS-222 (ethyl 3-aminobenzoate, Sigma-Aldrich, Saint Louis, MO, USA) for a minimum of 10 min following cessation of opercular movement to ensure death [37]. Once opercular movement decreased, the fish were transferred to the designated sample collection area. The fish were weighed using a DR-150 electronic hanging scale (Pesamatic, Santiago, Chile) and standard length was determined from the tip of the snout to the end of the caudal peduncle using an ichthyometer.

### 2.4. Blood Biochemistry Analysis

A blood sample of 10 mL was carefully collected from the caudal vein using a sterile syringe. The procedure was performed under appropriate conditions to prevent contamination. The samples were immediately transferred to anticoagulant-free tubes (BD Vacutainer^®^, Franklin Lakes, NJ, USA) and transported in a refrigerated container (4 °C) to the laboratory for analysis. The biochemical parameters analyzed included bilirubin, albumin, aspartate aminotransferase (AST), alanine aminotransferase (ALT), alkaline phosphatase (ALP), total proteins, gamma-glutamyl transferase (GGT), urea, phosphorus, calcium, creatinine, and globulins.

### 2.5. Semen Collection and Analysis

Semen was collected following the protocol described by Beirão et al. (2012) [45]. The urogenital pore was carefully dried, and semen was collected by abdominal massage (“stripping”). Samples contaminated with blood, urine, feces, or water were discarded. Semen was collected directly into a sterile, dry, and graduated plastic container maintained at 5 °C, allowing for ejaculate volume determination. Sperm concentration (millions of spermatozoa/mL) was determined using a Neubauer hemocytometer after diluting 1 μL of semen in 1200 μL of standard fish sperm medium (StorFish^®^, IMV Technologies, L’Aigle, France). Sperm concentration was measured in triplicate for each male.

### 2.6. Gonadosomatic Index (GSI)

Testes were carefully excised by dissection, rinsed with phosphate-buffered saline solution, and immediately weighed using a precision balance (Boeco, Hamburg, Germany). The GSI was calculated using the formula GSI = (testis weight × 100)/body weight [46].

### 2.7. Sperm Quality Analysis

Sperm motility was analyzed using a computer-assisted sperm analysis (CASA) system with AndroVision^®^ software version 1.1 (Minitüb GmbH, Tiefenbach, Germany). The percentage of motile spermatozoa was determined using a phase-contrast microscope (Zeiss AxioLab, Bovenden, Germany) at 100× magnification. Samples were activated using Powermilt^®^ at 280 mOs kg^−1^ and pH 9.0 (Universidad Católica de Temuco, Temuco, Chile), and a 0.25% *w*/*v* Pluronic solution (Sigma-Aldrich, Saint Louis, MO, USA) was added to prevent sperm adhesion to the slide. Activation and sequence recording were repeated at least five times. Sperm motility was assessed using fish sperm settings, with the following parameters: 25 frames s^−1^ at 50 Hz and an average trajectory velocity > 10 μm s^−1^ to classify sperm as motile.

Flow cytometry was used to determine plasma membrane integrity, mitochondrial membrane potential, and superoxide anion levels using a FACSCanto™ II flow cytometer (BD Biosciences, San Jose, CA, USA) with simultaneous excitation at 488 nm and 633 nm. Green fluorescence was measured in the FL-1 channel (480–530 nm), while red fluorescence was recorded in the FL-2 (580–630 nm) and FL-3 (610 nm) channels. Sample acquisition and analysis were performed using FACSDiva™ software version 6.1.3 (BD Biosciences, San Jose, CA, USA). Fluorescence compensation was applied using positive controls for each fluorochrome. Spectral overlap between fluorochromes was manually corrected by subtracting signals from each detector. A total of 10,000 events/sample were acquired at a rate of 600–1000 events s^−1^. Plasma membrane integrity was evaluated using the LIVE/DEAD sperm viability kit (Invitrogen Inc., Eugene, OR, USA), employing SYBR-14 and propidium iodide (PI) fluorescent probes. Mitochondrial membrane potential (ΔΨm) was assessed using the MitoProbe™ JC-1 Assay Kit (Invitrogen Inc., Eugene, OR, USA) with JC-1 and PI probes. Reactive oxygen species (ROS) production, specifically superoxide anion levels, was measured using dihydroethidium (DHE) and SYTOX Green probes (Invitrogen Inc., Eugene, OR, USA), following flow cytometry protocols described by Díaz et al. (2021) [47].

### 2.8. Fertilization Assay and Embryo Viability Assessment

Four males from each experimental group were selected to evaluate the effects of probiotics on sperm fertilization capacity. Eggs were obtained from mature Atlantic salmon (*Salmo salar*) females of the same genetic strain as the males, reared under identical hatchery conditions. The females (*n* = 10) were approximately 4–5 years old, with an average body weight of 7.73 ± 2.1 kg and a standard length of 77.6 ± 6.7 cm. The fish were euthanized as previously described, and the eggs were extracted via gentle abdominal massage. The eggs were maintained in ovarian fluid until fertilization. Fertilization batches were prepared by mixing eggs from ten females, divided into equal volumes (~10,000 eggs per batch). The fertilization trials were conducted in duplicate for each male, with a sperm concentration of 1.5 × 10^6^ spermatozoa per egg. Following fertilization, embryos were incubated in vertical flow hatching jars at 9 °C. Fertilization rates were calculated for all groups after 180 accumulated thermal units (ATUs), also known as degree days, which serve as a standardized measure for monitoring and comparing fish embryonic development based on cumulative temperature exposure over time. A sample of 100 eggs was taken from each batch and treated with a Stockard solution (BBC Biochemical, Mount Vernon, WA, USA) for 5 min. Each egg was then visually inspected, and the results were expressed as percentages. Eggs were considered fertilized if a formed embryo was visible. Infertile eggs showed no embryonic development, while dead eggs appeared white. Subsequently, embryo viability assessments were performed at 300 and 380 ATUs, recording the percentages of viable and non-viable (morphologically abnormal) embryos and mortality using stereoscopic microscopy (StereoBlue, Euromex, Duiven, The Netherlands).

### 2.9. Statistical Analysis

Statistical analysis was performed with Software R version 4.4.2 (2024) [48]. The results are expressed as the mean and standard error. The differences between groups were analyzed by Kruskal–Wallis due to the small size of the samples, and pairwise comparison was performed with Dunn’s test. The significance level was set at *p* < 0.05 using the rstatix package [49].

An ANCOVA test was performed to understand the effect of the total weight of the animals on the gonad weight. The total weight of the animals was included as a covariate using the following formula:

Weight_Gonads = β_0_ + β_1_ × Weight _total + β_2_ × Group + ϵ

A linear mixed-effects model was used to analyze the effect of treatment group on sperm viability over time, while controlling for the covariates of the sperm quality in fish (membrane integrity, high mitochondrial potential, anion superoxide negative, and total motility). The model included a random intercept for each fish to account for repeated measures over time. The analysis was performed using the lme4 package [50] in R. The model was specified as follows:

Viability = β_0_ + β_1_ × Group + β_2_ × ATUs + β_3_ × (Group × ATUs) + β_4_ × Mem + β_5_ × Mito + β_6_ × Anion + β_7_ × Motility + (1|ID/Group) + ϵ

where

Viability = the % viability of the spermatozoa;

Group = treatment group (A, B, or C);

ATUs = accumulated thermal units;

Mem = membrane integrity;

Mito = high mitochondrial potential;

Anion = anion superoxide negative;

Motility = total motility;

ID = fish identification number;

Beta0 = intercept;

Betai = coefficients;

epsilon = error term.

## 3. Results

### 3.1. Reproductive Parameters

Table 1 presents the results obtained, including both the mean and standard error (SE), regarding the effects of dietary supplementation with the probiotic *Pediococcus acidilactici* on the reproductive parameters of male Atlantic salmon (*Salmo salar*). In general, the biometric measurements, including body weight and standard length, showed no statistically significant differences among the experimental groups (*p* > 0.05), suggesting that the applied treatments did not produce detectable effects on the overall growth parameters or the body condition of the specimens. However, significant differences were found in the gonad weights among groups. Group C showed heavier gonads (0.28 ± 0.02 kg) compared to Group A (0.21 ± 0.03 kg) and Group B (0.16 ± 0.03 kg) (*p* = 0.048), while no significant differences were found between Groups A and B. The gonadosomatic index (GSI, %) was also significantly different between groups, with Group C showing the highest value (3.21 ± 0.21) compared to Group A (2.64 ± 0.18) and Group B (2.15 ± 0.18) (*p*-value = 0.028). Nevertheless, the GSI demonstrated a high variability without homogeneous variance (Levene’s test < 0.05).

To understand the effect of the total weight of the animals on the gonad weight, an ANCOVA test was performed (Table 2). When the total weight of the animals was included as a covariate, no significant difference in the gonad weight was found between groups (*p* > 0.05). The difference in the gonad weight found in Table 1 was explained by the total weight of the animals (*p* < 0.05) not by belonging to different groups (*p* > 0.05). Table 3 shows the adjusted means of the gonad weights by group accounting for the total weight of the animals.

### 3.2. Blood Biochemical Parameters

Table 4 shows the blood biochemistry analysis of the broodstock males (mean ± SE). Significant differences were found in the bilirubin and ALT levels among the groups. Bilirubin was significantly lower in Group C (1.98 ± 0.10) compared to Group B (2.45 ± 0.09) and Group A (2.00 ± 0.14) (*p*-value = 0.045). The ALT was significantly lower in Group C (12 ± 1) compared to Group B (17 ± 1) and Group A (24 ± 5) (*p*-value = 0.017). The other determined parameters showed no differences between the groups (*p* > 0.05).

### 3.3. Semen Quality Analysis

Table 5 shows the semen analysis of the three groups (mean ± SE), where only sperm concentration was significantly different between the groups. The sperm concentration (sperm/mL) was significantly lower in Group A (8.76 × 10^9^ ± 1.51 × 10^9^) compared to Group B (10.47 × 10^9^ ± 0.87 × 10^9^) and Group C (20.13 × 10^9^ ± 1.77 × 10^9^) (*p* < 0.001). No significant differences in sperm quality parameters, such as membrane integrity, percentage of sperm with high membrane mitochondrial potential, superoxide anion-negative cells, and total motility were observed (*p* > 0.05).

### 3.4. Fertilization and Embryo Viability

The fertility and embryo viability of the three groups are shown in Table 6, with data expressed as mean ± standard error (SE). Fertilization rates evaluated at 180 ATUs were significantly higher in Group C (59 ± 3) compared to Group B (50.0 ± 1), but no difference was observed with Group A or between Groups A and B. Moreover, a significant decrease in non-fertilized eggs was observed in Group C (120 days of supplementation with *Pediococcus acidilactici*) compared to Group A (control), while no significant differences were found between Groups A and B or between B and C. No significant differences in mortality rates were observed in this stage.

**Figure 1:**
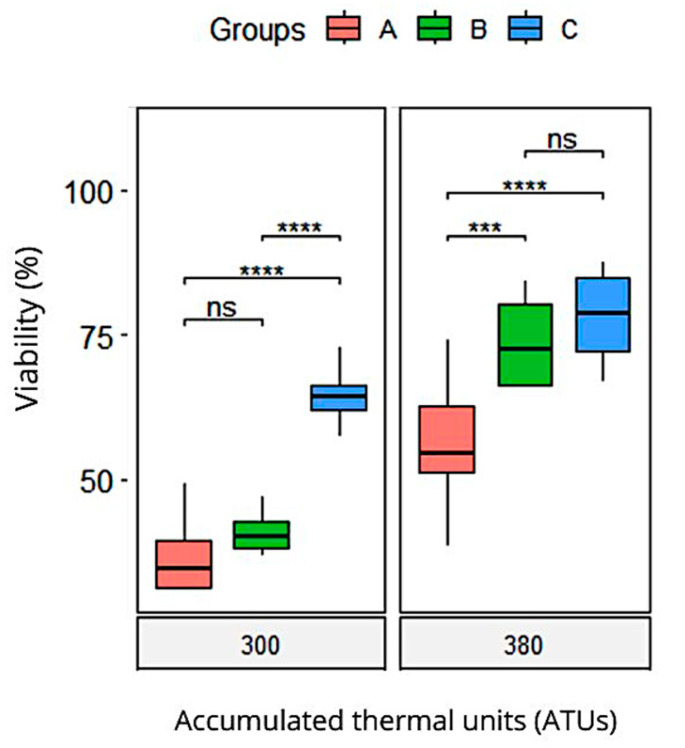
Boxplots of viability of *Salmo salar* offspring embryos supplemented with *Pediococcus acidilactici.* Significant differences are indicated (ns *p* > 0.05; *** *p* < 0.001; **** *p* < 0.0001). Group A: breeding males without probiotic supplementation (control group); Group B: breeding males supplemented with the probiotic *Pediococcus acidilactici* for 60 days; Group C: breeding males supplemented with the probiotic *Pediococcus acidilactici* for 120 days.

The embryonic viability rate was significantly higher in Group C at both 300 ATUs and 380 ATUs (Figure 1). At 300 ATUs, Group C exhibited an average viability rate of 64 ± 2%, whereas Groups A and B recorded similar mean values of 36.7 ± 2% and 41.1 ± 1%, respectively. At 380 ATUs, the percentage of viable embryos without evident morphological alterations in Group C was 78.1 ± 3%. Similarly, Group B showed a high viability rate of 73.6 ± 3%, with no significant differences between the two groups. Meanwhile, Group A achieved an embryonic viability rate of 56.7%, with statistically significant differences compared to Groups B and C.

**Figure 2:**
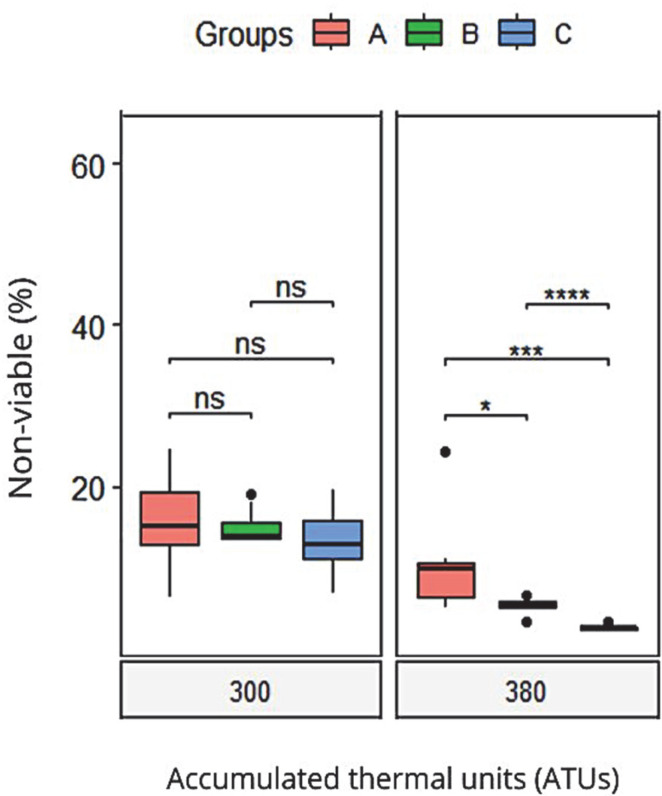
Boxplots of non-viable embryos offspring of *Salmo salar* supplemented with *Pediococcus acidilactici*. Significant differences are indicated (ns *p* > 0.05; * *p* < 0.05; *** *p* < 0.001; **** *p* < 0.0001). Group A: breeding males without probiotic supplementation (control group); Group B: breeding males supplemented with the probiotic *Pediococcus acidilactici* for 60 days; Group C: breeding males supplemented with the probiotic *Pediococcus acidilactici* for 120 days.

Additionally, at 300 ATUs, no significant differences were found between the three groups concerning the percentage of non-viable embryos. In contrast, at 380 ATUs, both Group B (22.1 ± 2) and Group C (20.5 ± 3) showed the lowest malformation rates (non-viable embryos), while Group A had a higher incidence of malformations at 34.1 ± 4%, with significant differences compared to Groups B and C (Figure 2). These results suggest that probiotic supplementation with *Pediococcus acidilactici* can reduce non-viability, especially in advanced developmental stages.

**Figure 3:**
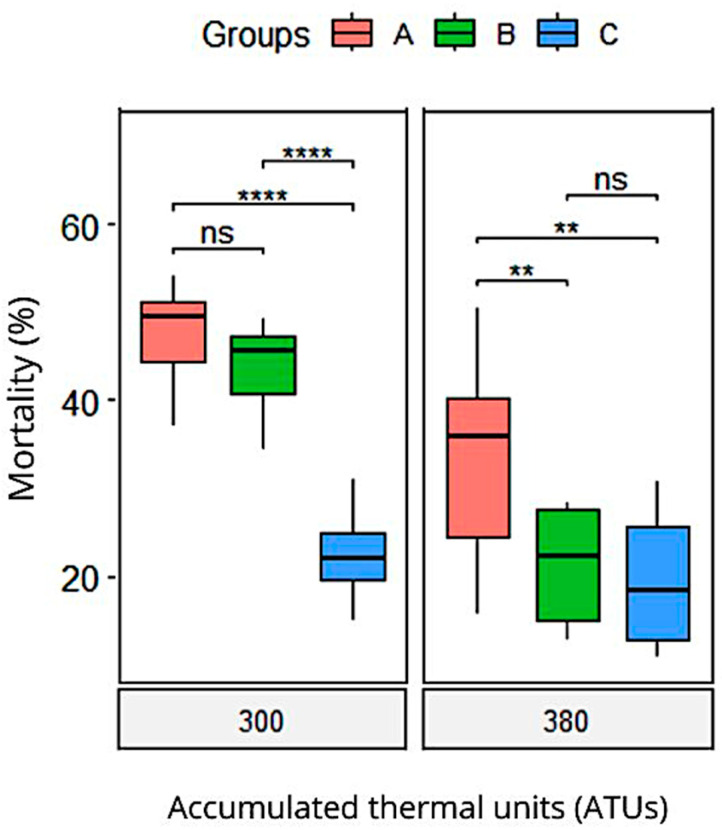
Boxplots of mortality of *Salmo salar* offspring embryos supplemented with *Pediococcus acidilactici*. Significant difference is indicated (ns *p* > 0.05; ** *p* < 0.01; **** *p* < 0.0001). Group A: breeding males without probiotic supplementation (control group); Group B: breeding males supplemented with the probiotic *Pediococcus acidilactici* for 60 days; Group C: breeding males supplemented with the probiotic *Pediococcus acidilactici* for 120 days.

Finally, we observed that at 300 ATUs, Group C (23.2 ± 2) had a significantly lower embryonic mortality rate compared to Groups A (48.3 ± 2) and B (44.7 ± 2), with no significant differences between the latter two groups. At 380 ATUs, both Groups B (4.3 ± 0.38) and C (1.46 ± 0.14) showed significantly lower mortality compared to Group A (9.4 ± 2.2), with no differences between Groups B and C (Figure 3). These findings indicate that supplementation with *Pediococcus acidilactici*, especially for 120 days, reduces embryonic mortality.

A linear mixed-effects model was used to analyze the effect of the treatment group on viability over time, while controlling for the covariates of the sperm quality in Atlantic salmon (membrane integrity, high membrane mitochondrial potential, anion superoxide negative, and total motility). The model included a random intercept for each fish to account for repeated measures over time. The results of the model are presented in Table 7. The analysis revealed significant main effects of time (ATUs) and treatment group. The interaction between time and treatment group was also significant, indicating that changes in viability over time varied by treatment group. None of the covariates were significant predictors of viability (all *p* > 0.05). Between the groups, the contrast analysis indicates a better performance for treatment groups B and C compared to the control group A in the group vs. ATU interaction. As a group variable itself, the C treatment had a higher viability than the B treatment.

## 4. Discussion

Probiotics are widely utilized in animal production, including aquaculture, to enhance growth, immunity, and health, representing a sustainable alternative to antibiotics [1,20,22,24,51,52,53,54,55,56]. In fish, probiotic supplementation improves digestion, nutrient assimilation, and water quality management [57]. Furthermore, probiotics have demonstrated the potential to enhance reproductive performance such as fertility and embryo survival by modulating intestinal microbiota and the activation of key hormones and enzymes [24,58,59,60]. Probiotics are commonly delivered through feed, water, or injections, and their combination with plant-based diets has been shown to promote sustainability in aquaculture systems [61,62]. Although the positive effects of probiotics on fish health are well documented, their role in reproduction, particularly in salmonids, is still being explored.

In this context, the present study examined the effects of probiotic supplementation on reproductive parameters in male Atlantic salmon. We observed that there were no significant differences in final weight among the dietary treatments (Table 1), which may be attributed to the advanced stage of sexual maturation in the specimens, a period typically characterized by reduced feed intake during the final weeks of the trial. Similarly, Ferguson et al. (2010) [63] did not detect significant differences in the productive parameters of *Oreochromis niloticus* fed a diet supplemented with *Pediococcus acidilactici* at a concentration of 10^7^ CFU g^−1^ for 32 days. However, their research showed that supplementation with this probiotic modulated the intestinal bacterial communities of growing *O. niloticus* and stimulated certain aspects of the non-specific immune response, which was not addressed in this study. On the other hand, studies with other probiotics, such as *Bacillus amyloliquefaciens* AV5, showed contrasting results. In a study conducted on Nile tilapia (*O. niloticus*), fish fed a diet supplemented with the highest concentration of this probiotic (10^8^ CFU/g) experienced significant improvements in growth parameters, including final weight, specific growth rate, and weight gain rate, compared to the control group without supplementation. These results highlight that the effects of probiotics on growth and maturation in fish may vary depending on the species, the type of probiotic, and the experimental conditions. Nevertheless, further research is needed to determine whether the increase in gonad size observed in Group C (120 days of supplementation) is attributable exclusively to the administration of the probiotic *Pediococcus acidilactici* or to a potential interaction with the maturation time [64].

In this study, the supplementation of the salmonid diet with *Pediococcus acidilactici* showed a significant increase in reproductive parameters such as gonadal weight, gonadosomatic index, and sperm concentration in experimental Group C, suggesting a positive effect of the probiotic on the sexual maturation of salmonids. These findings are consistent with those reported by Abdelal et al. (2021) [65], who evaluated the impact of dietary supplementation with the probiotic Bactocill (*P. acidilactici*) in *Oreochromis niloticus* (Nile tilapia), observing significant improvements in gonadal weight, gonadosomatic index, and in the volume and concentration of fresh semen during two reproductive seasons. Similarly, previous studies on *Danio rerio* (zebrafish) fed a diet containing 4 × 10^6^ CFU of *P. acidilactici* per gram of food reported a significant increase in gonadal weight, gonadosomatic index, fresh semen volume, and sperm concentration compared to the control group (*p* < 0.05), indicating that this probiotic dose is effective in optimizing reproductive performance in zebrafish [38]. In addition, Rahman et al. (2018) [66] demonstrated that a probiotic-enriched diet significantly improved the gonadosomatic index in *Ompok pabda* (Pabda catfish) larvae over a 60-day period, supporting the effectiveness of probiotic strategies in enhancing reproductive performance. Carnevali et al. (2017) [67] also documented the beneficial effects of probiotics on fish gonads, particularly in zebrafish, highlighting their influence on reproduction and gamete quality. Furthermore, studies conducted in *O. niloticus* and *Oncorhynchus mykiss* have shown that probiotics contribute to the stabilization of the intestinal microbiota, which in turn supports reproductive processes through the regulation of related hormones and enzymes [58,59].

In this regard, previously described results on the effects of dietary supplementation with the probiotic *Bacillus amyloliquefaciens* AV5 in *Oreochromis niloticus* (Nile tilapia) showed a notable increase in gonadal weight, gonadosomatic index, fresh semen volume, and sperm concentration in the treatment groups that received the diet enriched with this probiotic [64]. Similarly, Mehdinejad et al. (2019) [39] reported that the probiotic *P. acidilactici* can improve the reproductive performance of goldfish (*Carassius auratus*) by favoring gonad size and function. They also highlighted that probiotics optimize feed efficiency, nutrient absorption, and immune response, which indirectly supports gonadal development by ensuring greater nutrient availability for reproduction. Additionally, Arani et al. (2021) [38] investigated the effect of different levels of *Pediococcus acidilactici*, including 0 (basal diet as control), 1 × 10^6^, 2 × 10^6^, 4 × 10^6^, and 8 × 10^6^ CFU g^−1^ of diet for 60 days on growth and reproductive performance in zebrafish (*Danio rerio*). The results showed that the best growth and reproductive indices were associated with a concentration of 4 × 10^6^ CFU g^−1^ (*p* < 0.05). Furthermore, the gonadosomatic index increased proportionally with this concentration of the probiotic, consistent with the increase in weight and the changes observed in the gonads of the salmon fed for longer periods in this study.

It has been shown that beneficial microorganisms optimize the utilization of energy sources derived from the diet, thereby promoting growth [68,69,70]. This positive effect is attributed to the ability of probiotics to produce enzymes, vitamins, and other substances beneficial to the host [71], as well as stimulating their endogenous secretion, thus improving digestion and nutrient absorption. Mehdinejad et al. (2019) [39] evaluated the effects of the probiotic *P. acidilactici* and nucleotide supplementation, both individually and in combination, on the hematological parameters of goldfish (*Carassius auratus*). In particular, the combination of 0.2% probiotic and 0.5% nucleotides resulted in the highest values of hemoglobin, hematocrit, total protein, glucose, albumin, and globulin. Additionally, the highest red and white blood cell counts were recorded in fish fed with 0.3% probiotic, either alone or in combination with 0.5% nucleotides. Furthermore, Dhanasiri et al. (2023) [28] investigated the effect of diets supplemented with functional ingredients on intestinal health and performance in Atlantic salmon (*Salmo salar*) during the post-smolt phase. Fish fed a diet enriched with fructo-oligosaccharides and *P. acidilactici* showed significantly superior growth compared to the control group, with thermal growth coefficients of 3.23 and 2.96, respectively. Although the studies by Mehdinejad et al. (2019) [39] and Dhanasiri et al. (2023) [28] were of shorter duration than the present study, their findings align with our results, as the inclusion of *P. acidilactici* in the salmonid diet did not negatively alter the blood biochemical profile, which could positively affect health and reproductive performance.

In a previous study, Eissa et al. (2022) [72] investigated the influence of probiotic supplementation in the diet of European bass (*Dicentrarchus labrax*) and observed that the variability of results could be related to the fish’s biochemical profile. Specifically, blood parameters such as total proteins, albumin, and glucose levels showed significant differences between groups fed different doses of probiotics. These biochemical changes reflect how the inclusion of probiotics in the diet can affect the nutritional profile and intestinal health of fish. Additionally, improvements in body composition and growth rates support the hypothesis that the biochemical profile plays a crucial role in the physiological response of fish to feeding and environmental conditions. However, in our study, no significant differences were detected in the blood biochemical profile. Although variations in bilirubin and ALT levels were observed (Table 4), these values were within the normal reference ranges for the species (Bilirubin: <4.4 μmol/L; ALT: <24 U/L), suggesting that the changes observed were not physiologically significant and likely reflect normal biological variability.

Reproductive success in fish is closely linked to energy balance, as metabolic state influences the hormonal and molecular regulation of reproductive functions through the hypothalamic-pituitary-gonadal axis [14,73]. This regulation is especially important in captivity, where reproductive performance is crucial for growth, development, and the success of breeding programs [1]. The reproductive improvements observed in our study, particularly the increase in sperm concentration (Table 5), align with previous findings in zebrafish, where supplementation with *Pediococcus acidilactici* modulated the expression of genes involved in spermatogenesis and enhanced testicular structure [74,75]. These findings support the hypothesis that probiotic supplementation can modulate reproductive physiology through metabolic and molecular pathways, contributing to improved fertility outcomes in aquaculture species.

This hypothesis is further substantiated by studies involving other probiotic strains. For example, *Lactobacillus rhamnosus* has been reported to improve fertility, gamete quality, and reproductive performance in zebrafish by regulating genes associated with ovulation and follicular development, resulting in increased ovulated egg numbers and enhanced embryonic viability [37]. In addition, Valcarce et al. [74] demonstrated that dietary supplementation with *L. rhamnosus* and *Bifidobacterium longum* significantly improved sperm concentration, motility, and the proportion of fast-swimming sperm within a 21-day period, corresponding to a complete spermatogenic cycle. These effects have been attributed to the stimulation of Leydig cells, enhancement of Sertoli cell function, and support of vascular and immune components essential for testicular function [76]. In European eels, administration of *L. rhamnosus* (10^5^ CFU/mL) for two weeks led to increased sperm output and motility, concomitant with upregulation of activin, androgen receptors (AR), and follicle-stimulating hormone receptors (*fshr*), genes critically involved in spermatogenesis [36]. At the molecular level, probiotics have also been linked to the upregulation of testicular genes involved in spermatogenic regulation, including leptin, brain-derived neurotrophic factor (*bdnf*), and doublesex and mab-3-related transcription factor 1 (*dmrt1*) [36]. Moreover, the increased transcriptional activity of activin, ARα, ARβ, progestin receptor 1 (*pr1*) and *fshr* has been associated with improved sperm quality during spermatogenesis. These molecular responses underscore the potential of probiotics to optimize testicular function and reproductive health in fish. However, the present study did not evaluate the expression of reproductive genes. Future investigations should therefore focus on elucidating the specific effects of *P. acidilactici* on gene expression in *Salmo salar*, to clarify its mechanism of action and support its application in reproductive management strategies for aquaculture.

On the other hand, Mehdinejad et al. (2019) [39] evaluated the effect of dietary supplementation with different concentrations of *Pediococcus acidilactici* and nucleotides on the reproductive performance of goldfish (*Carassius auratus*) over 180 days. The study analyzed various reproductive parameters, including semen quality (motility and sperm density) and egg quality (egg diameter, absolute and relative fecundity, gonadosomatic and hepatosomatic indices, fertilization rate, and hatchability). The results indicated that supplementation with probiotics and nucleotides significantly improved semen quality and reproductive indices compared to the control diet. In particular, the addition of 0.3% probiotic resulted in higher sperm density and a significant increase in sperm index. On the other hand, females fed enriched diets showed substantial improvements in fecundity indices and hatchability rates. Similarly, males receiving a combined diet with 0.2% probiotics and nucleotides exhibited the highest percentage and duration of sperm motility, as well as the highest absolute fecundity and fertilization rate.

In the present study, a higher proportion of viable embryos was observed from male breeders who received a probiotic diet compared to the control group during the early stages of embryonic development (Table 6). It has been demonstrated that probiotics can improve embryonic quality in fish by optimizing the chemical composition of oocytes during vitellogenesis and regulating the expression of genes associated with reproduction, which translates into higher fertilization rates and healthier embryos [77]. In particular, the *Lactobacillus rhamnosus* IMC 501 probiotic has shown positive effects on ovarian development in female zebrafish. The administration of *L. rhamnosus* at a concentration of 10^6^ CFU/g improved fecundity, gonadosomatic index, and oocyte maturation, both during advanced vitellogenesis and final maturation, thus contributing to higher reproductive performance [37].

The findings of this study demonstrate a significant reduction in the incidence of embryonic malformations (non-viable embryos) at 380 ATUs in the offspring of groups supplemented with *Pediococcus acidilactici* (Table 6), alongside improved embryo viability—particularly in group C, which exhibited the highest viability among treatments (Table 7). These results align with previous studies, such as that of Ghosh et al. (2007) [46], who conducted a comprehensive investigation into the effects of probiotic supplementation with *Bacillus subtilis* in female breeders of four ornamental fish species (*Poecilia reticulata*, *Poecilia sphenops*, *Xiphophorus helleri*, and *Xiphophorus maculatus*). In their study, *B. subtilis* was shown to significantly improve reproductive parameters, including fecundity, fry production, and fry growth (length and weight). Importantly, their research also highlighted a marked reduction in fry mortality and a notable decrease in the incidence of malformations in groups fed probiotic-supplemented diets. This suggests that probiotics can play a crucial role in enhancing both the survival and overall development of offspring. The improvements in reproductive outcomes highlight probiotics as a promising functional additive in aquaculture. Their ability to enhance immunity, reduce mortality, and decrease antibiotic reliance is increasingly relevant for sustainable animal production. Specifically, *P. acidilactici* supplementation in Atlantic salmon diets may improve reproductive performance and fry viability, benefiting animal welfare, product quality, and environmental sustainability. These findings underscore the potential of probiotics to optimize productivity in aquaculture systems.

## 5. Conclusions

The results of this study confirm that prolonged dietary supplementation with *Pediococcus acidilactici* has a positive impact on reproductive performance in *Salmo salar*, evidenced by significant improvements in semen quality, higher fertilization rates, and more efficient embryo development. These effects suggest that this probiotic may play a key role in optimizing reproductive processes in species of aquaculture interest, contributing to more efficient and sustainable production. Moreover, these findings establish *Pediococcus acidilactici* as a viable strategy for improving reproductive performance in aquaculture. However, to fully understand its potential and its large-scale applications, it is essential to carry out further research that explores its mechanisms of action at the molecular level, its interaction with the gut microbiota and its long-term impact on the reproductive physiology of different aquaculture species. The incorporation of this probiotic in reproductive management programs could make a significant difference in the sustainability and efficiency of the industry, opening new opportunities for a more efficient and productive aquaculture.

## Figures and Tables

**Table 1:** Analysis of reproductive parameters in *Salmo salar* supplemented with *Pediococcus acidilactici*, including total weight (kg), standard length (cm), gonad weight (kg), gonadosomatic index (GSI, %), and fresh semen volume (mL).

Parameter	A N = 16	B N = 16	C N = 16	*p*-Value
Total weight (kg)	7.71 (0.61)	7.30 (1.07)	8.33 (0.51)	0.22
Standard length (cm)	79.2 (1.9)	77.6 (2.3)	81.0 (2.0)	0.59
Gonad weight (kg)	0.21 (0.03) ^a^	0.16 (0.03) ^a^	0.28 (0.02) ^b^	0.048
GSI (%)	2.64 (0.18) ^ab^	2.15 (0.18) ^a^	3.21 (0.21) ^b^	0.028
Fresh semen volume (mL)	12.0 (2.5)	20.0 (0.0)	14.9 (1.8)	0.21

^a,b^ Means (std. error) in the same row with different superscripts differ significantly (pairwise Dunn’s test *p* < 0.05). Group A: breeding males without probiotic supplementation (control group); Group B: breeding males supplemented with the probiotic *Pediococcus acidilactici* for 60 days; Group C: breeding males supplemented with the probiotic *Pediococcus acidilactici* for 120 days.

**Table 2:** ANCOVA test of the gonad weight (kg) by group (A, B, and C) accounting for the total weight of the animals (kg).

	Sum Sq	Df	F Value	Pr (>F)
(Intercept)	0.00	1	0.04	0.84
Total weight	0.07	1	16.32	0.00
Group	0.02	2	2.47	0.11
Residuals	0.09	21		

Group A: breeding males without probiotic supplementation (control group); Group B: breeding males supplemented with the probiotic *Pediococcus acidilactici* for 60 days; Group C: breeding males supplemented with the probiotic *Pediococcus acidilactici* for 120 days.

**Table 3:** Adjusted means of the gonad weight (kg) by group (A, B, and C) accounting for the total weight of the animals (kg).

Group	Adj. Mean	CI 95%	CI 95%
A	0.22	0.16	0.29
B	0.19	0.12	0.26
C	0.26	0.23	0.30

Group A: breeding males without probiotic supplementation (control group); Group B: breeding males supplemented with the probiotic *Pediococcus acidilactici* for 60 days; Group C: breeding males supplemented with the probiotic *Pediococcus acidilactici* for 120 days.

**Table 4:** Blood biochemistry analysis of the *Salmo salar* supplemented with *Pediococcus acidilactici*.

Parameter	A N = 16	B N = 16	C N = 16	*p*-Value
Bilirubin (μmol/L)	2.00 (0.14) ^ab^	2.45 (0.09) ^a^	1.98 (0.10) ^b^	0.045
Albumin (g/L)	19.50 (0.65)	20.00 (1.08)	20.57 (0.36)	0.42
AST (U/L)	228 (17)	234 (11)	229 (8)	0.82
ALT (U/L)	24 (5) ^a^	17 (1) ^ab^	12 (1) ^b^	0.017
Alkaline phosphatase (U/L)	972 (196)	900 (110)	992 (67)	0.93
Protein (g/L)	55.8 (2.3)	58.0 (1.3)	57.8 (1.5)	0.74
GGT	0.75 (0.48)	1.25 (0.75)	1.36 (0.32)	0.68
Urea (mmol/L)	1.00 (0.14)	1.08 (0.05)	1.01 (0.03)	0.52
Phosphorus (mmol/L)	5.35 (0.07)	5.33 (0.14)	5.41 (0.05)	0.74
Calcium (mmol/L)	3.14 (0.06)	3.24 (0.08)	3.20 (0.05)	0.65
Creatinine (μmol/L)	13.7 (2.6)	14.1 (0.7)	16.2 (1.7)	0.47
Globulins (g/L)	36.3 (1.9)	38.0 (0.7)	37.2 (1.3)	0.69

^a,b^ Means (std. error) in the same row with different superscripts differ significantly (pairwise Dunn’s test *p* < 0.05). Aspartate aminotransferase (AST), alanine aminotransferase (ALT), alkaline phosphatase (ALP), and gamma-glutamyl transferase (GGT). Group A: breeding males without probiotic supplementation (control group); Group B: breeding males supplemented with the probiotic *Pediococcus acidilactici* for 60 days; Group C: breeding males supplemented with the probiotic *Pediococcus acidilactici* for 120 days.

**Table 5:** Semen quality analysis of the *Salmo salar* supplemented with *Pediococcus acidilactici*.

Characteristic	A N = 16	B N = 16	C N = 16	*p*-Value
Sperm concentration (sperm/mL)	8.76 × 10^9^ (1.51 × 10^9^) ^a^	10.47 × 10^9^ (0.87 × 10^9^) ^a^	20.13 × 10^9^ (1.77 × 10^9^) ^b^	<0.001
Membrane integrity (%)	97.83 (0.39)	97.83 (0.24)	98.06 (0.31)	0.76
Membrane mitochondrial potential (%)	62 (6)	64 (4)	63 (5)	0.89
Anion superoxide negative (%)	95.75 (0.62)	95.96 (0.60)	96.38 (0.24)	0.90
Total motility (%)	79 (3)	85 (3)	84 (3)	0.28

^a,b^ Means (std. error) in the same row with different superscripts differ significantly (pairwise Dunn’s test *p* < 0.05). Group A: breeding males without probiotic supplementation (control group); Group B: breeding males supplemented with the probiotic *Pediococcus acidilactici* for 60 days; Group C: breeding males supplemented with the probiotic *Pediococcus acidilactici* for 120 days.

**Table 6:** Fertility and offspring viability evaluation of the *Salmo salar* supplemented with *Pediococcus acidilactici*.

Stage	Characteristic	A N = 8	B N = 8	C N = 8	*p*-Value
Fertility (%)					
180 ATUs	Fertilized eggs	51.2 (3) ^ab^	50.0 (1) ^a^	59.1 (3) ^b^	0.039
	Non-fertilized eggs	28.5 (2.4) ^a^	24.2 (0.6) ^ab^	20.1 (1.1) ^b^	0.004
	Mortality	20.3 (2.1)	25.8 (1.0)	20.8 (2.2)	0.14
Embryo viability (%)					
300 ATUs	Viability	36.7 (2) ^a^	41.1 (1) ^a^	64.4 (2) ^b^	<0.001
	Non-viable	15.0 (2.2)	14.2 (0.8)	12.4 (1.5)	0.47
	Mortality	48.3 (2) ^a^	44.7 (2) ^a^	23.2 (2) ^b^	<0.001
380 ATUs	Viability	56.5 (4) ^a^	73.6 (3) ^b^	78.1 (3) ^b^	0.003
	Non-viable	34.1 (4) ^a^	22.1 (2) ^b^	20.5 (3) ^b^	0.035
	Mortality	9.4 (2.2) ^a^	4.3 (0.38) ^b^	1.4 (0.14) ^b^	<0.001

^a,b^ Means (std. error) in the same row with different superscripts differ significantly (pairwise Dunn’s test *p* < 0.05). Group A: breeding males without probiotic supplementation (control group); Group B: breeding males supplemented with the probiotic *Pediococcus acidilactici* for 60 days; Group C: breeding males supplemented with the probiotic *Pediococcus acidilactici* for 120 days. ATUs: accumulated thermal units.

**Table 7:** The results of the linear mixed-effects model examining the effect of treatment group on viability over time, while controlling for the covariates of sperm quality in fish.

Characteristic	Contrast	Estimate	Std. Error	95% CI	*p*-Value
(Intercept)		40	91.76	−140, 220	0.67
Group					0.002
	A × (B + C)	1.3	0.82	−0.36, 2.9	0.13
	B × C	4.5	1.39	1.7, 7.2	0.003
ATUs					<0.001
	300	−6.0	1.39	−8.7, −3.3	<0.001
	380	16	1.39	13, 19	<0.001
Mem		0.21	1	−1.8, 2.2	0.84
Mito		0.01	0.06	−0.10, 0.13	0.83
Anion		0.00	0.49	−0.97, 0.98	>0.99
Motility		−0.10	0.11	−0.32, 0.13	0.40
Group × ATUs					<0.001
ATUs 300	A × (B + C)	4.2	0.97	2.3, 6.2	<0.001
ATUs 300	B × C	7.1	1.69	3.8, 10	<0.001
ATUs 380	A × (B + C)	5.3	0.97	3.4, 7.3	<0.001
ATUs 380	B × C	−2.3	1.69	−5.6, 1.0	0.17

Group A: breeding males without probiotic supplementation (control group); Group B: breeding males supplemented with the probiotic *Pediococcus acidilactici* for 60 days; Group C: breeding males supplemented with the probiotic *Pediococcus acidilactici* for 120 days. ATUs: accumulated thermal units.

## Data Availability

All data are included within the manuscript.
